# Supplementary material for: De novo evolved interference competition promotes the spread of biofilm defectors
Source: Nat Commun. 2017 May 2;8:15127. doi: 10.1038/ncomms15127 (PMC5418572; doi:10.1038/ncomms15127)
Supplement: Supplementary Information — Supplementary Figures, Supplementary Tables and Supplementary References [file ncomms15127-s1.pdf]

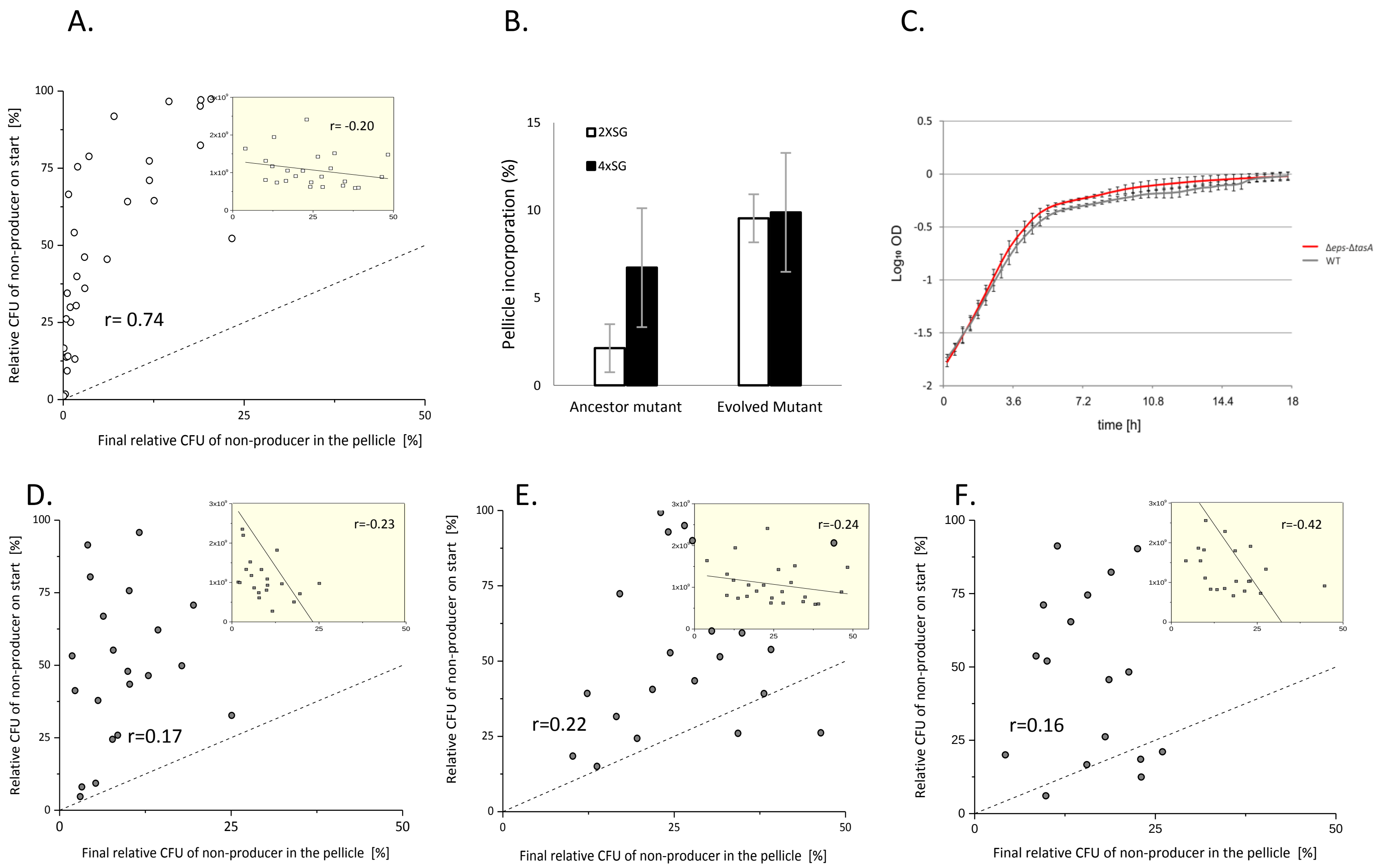

Supplementary Figure 1. Pellicle competition assays at different starting ratio of the producer and non-producer. A. Pellicle competition assay of *B. subtilis* wild type against the non-producer *Δeps-ΔtasA* at different starting (%) ratio (n=28; r=0.74). The incorporation of the non-producer *Δeps-ΔtasA* into the pellicle is dependent on its initial frequency. Dashed line represents 1.0 slope. Total CFU in the pellcle for each final relative CFU on the non-producer were plotted in the upper-right corner. **B.** Pellicle competition assay of *B. subtilis* wild type against the non-producer Δ*eps*-Δ*tasA* (left: ancestor, right: evolved) at different nutrient concentrations (n=5): 2xSG medium (standard nutrient concentration) and 4xSG medium (doubled nutrient concentration). Starting ratios were set to 50%. Pellicle incorporation of the ancestor non-producer Δ*eps*-Δ*tasA* escalates as the nutrient concentration increases. On the contrary, nutrient concentration has no effect on incorporation of the eNMP into the pellicle. Values represent the mean and error bars s.d.m. **C.** Growth curve of the *B. subtilis* 168 wild-type and Δ*eps*-Δ*tasA* mutant. OD^{595} was measured every 15 min using a TECAN Infinite F200 PRO microplate reader. The experiment was conducted in 2xSG medium at 30°C (n=9). Central values represent the mean and error bars s.d.m. Calculated growth rates [OD/h] for the WT and Δ*eps*-Δ*tasA* were μ=0.65 and μ=0.71, respectively, indicating a faster growth of the mutant strain; *t*-Student, two-tail $p < 0.05$. **D-F.** Pellicle incorporation of evolved non-producers at different starting (%) ratios. The incorporation success of the evolved non-producers B310mA (n=21; r=0.17) (**D**), B410mB (n=21; r=0.22) (**E**) and B510mC (n=18; r=0.16) (**F**) does not depend on their initial frequency. Dashed lines represent 1.0 slopes. Total CFU in the pellcle for each final relative CFU on the non-producer were plotted in the upper-right corner in each graph. Each experiment was performed at least twice.

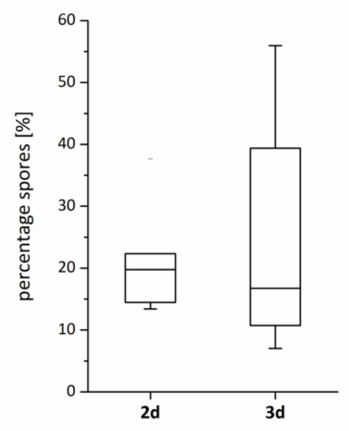

Supplementary Figure 2. Sporulation frequency in *B. subtilis* 168 pellicle. Sporulation frequency in *B. subtilis* 168 pellicle was determined after 2 and 3 days by comparing the total CFU (vegetative cells+spores) with the CFU after 20 min of incubation at 80°C (where only the spores survive) (n=7). Boxes represent Q1-Q3, lines represent the median and bars span from max to min. The experiment performed twice.

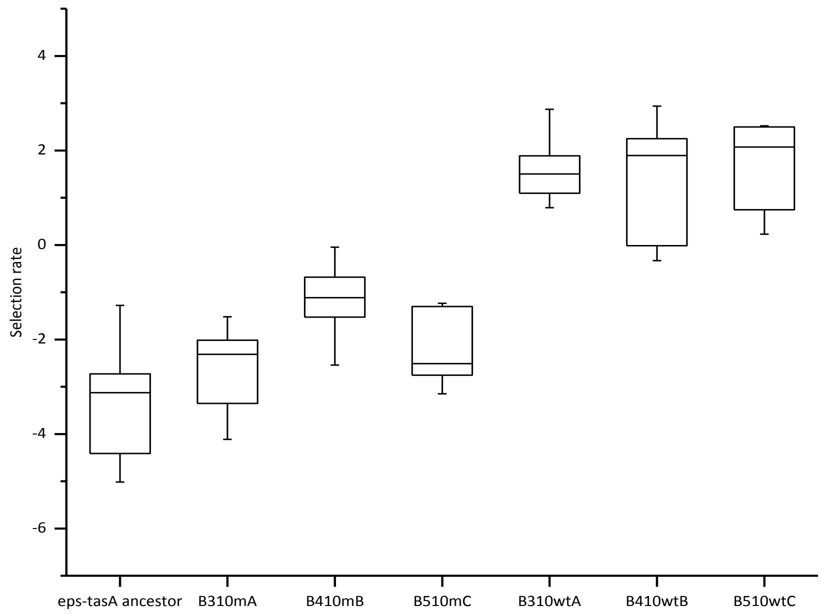

Supplementary Figure 3. Improved selection rate of evolved non-producers and producers against the wild type ancestor. Performance of selected strains against the wild type ancestor in pellicle competition assay was examined and the selection rate for each strain was calculated. The initial ratios of the strains in competition assays were in the range from 35% to 70% (n = 6-12). Boxes represent Q1-Q3, lines represent the median and bars span from max to min. Each *Δeps-ΔtasA* vs eMP/eNMP comparison was performed at least twice. The incorporation of the eMPs B310wtA, B410wtB and B510wtC into pellicles were 73.2 ±6.4(%), 67.8 ±8.8(%) and 82.0 ±2.9(%), respectively.

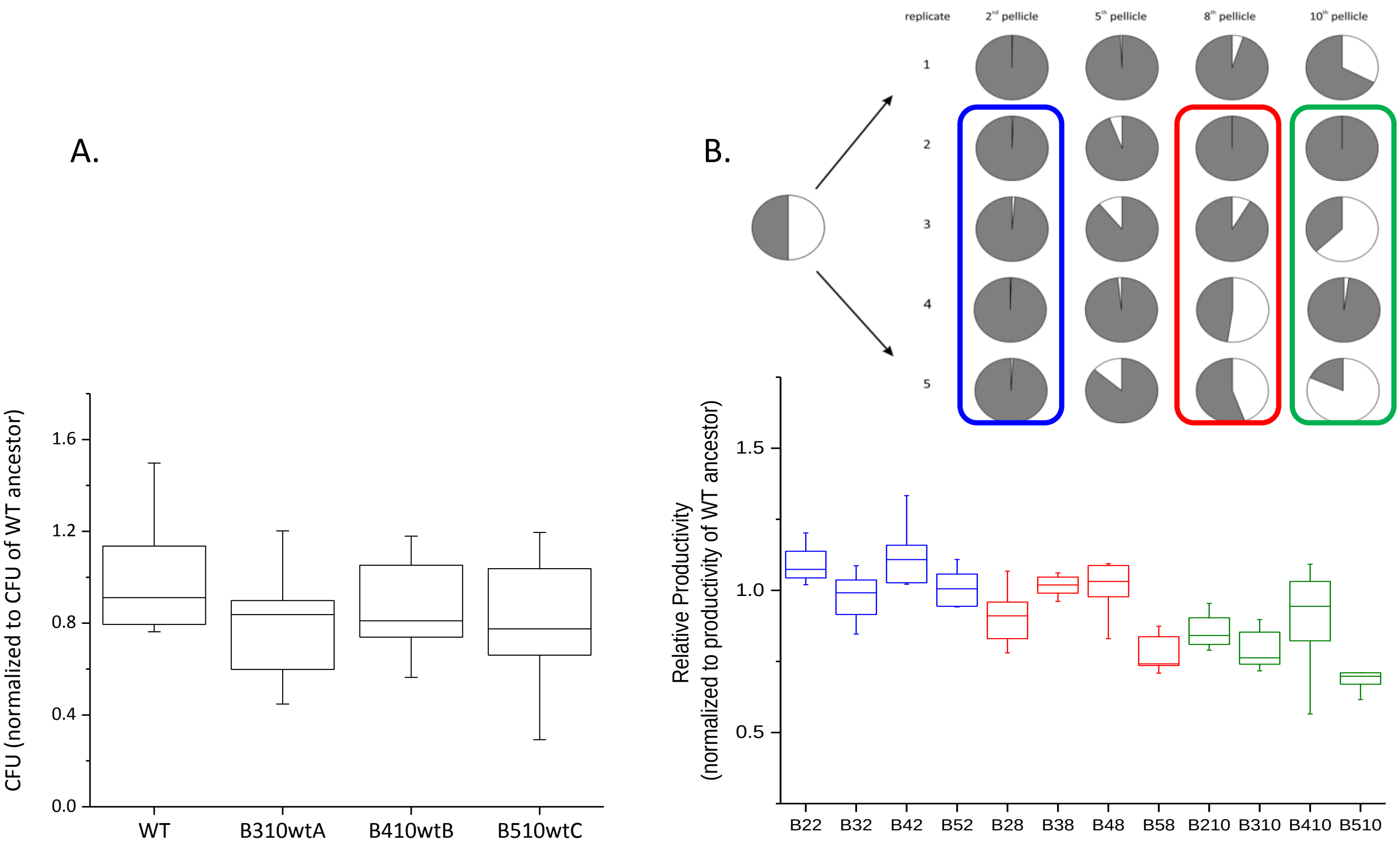

Supplementary Figure 4. Viable cell counts and productivity changes in the evolved pellicles. A. Total cell count of pellicles of evolved WT strains, B310wtA, B410wtB, B510wtC (n=10) normalized to the CFU of WT ancestor showing no significant difference compared to WT ancestor. We did not observe increased viable cell numbers in the eMP, as was the case for the overall biomass. We hypothesize that increased frequency of cell lysis, linked to spontaneous lytic induction in the eMP, may lead to release of various substances that contribute to the extracellular matrix and overall biomass. **B.** Productivity estimates of pellicle population mixes of WT and Δ*eps*-Δ*tasA* in replicates 2, 3, 4 and 5 at different time points (2nd, 8th, and 10th transfer) (n=10); Boxes represent Q1-Q3, lines represent the median and bars span from max to min. The experiments were performed twice.

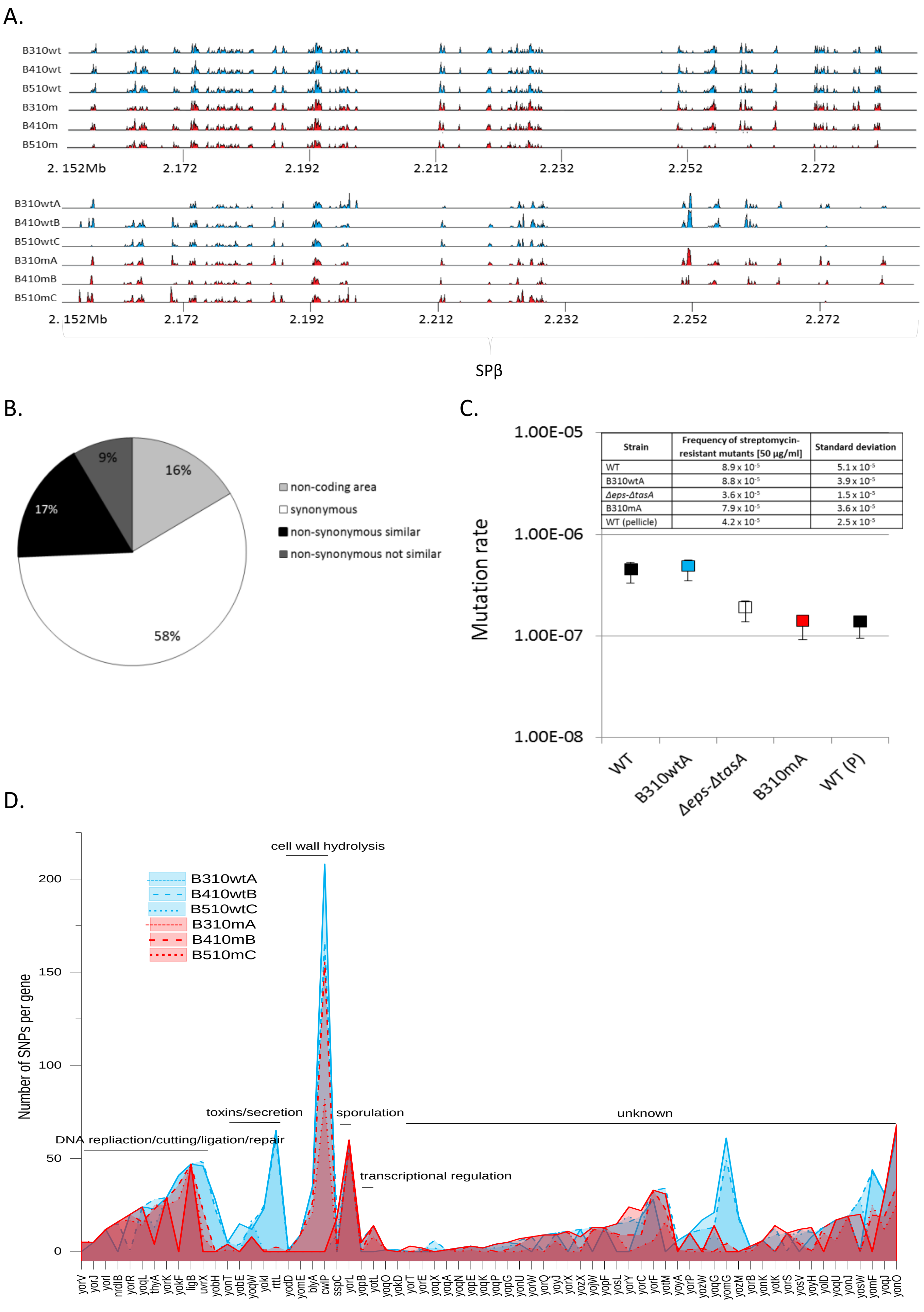

Strain	Frequency of streptomycin-resistant mutants [50 μg/ml]	Standard deviation
WT	8.9×10^{-5}	5.1×10^{-5}
B310wtA	8.8×10^{-5}	3.9×10^{-5}
Δeps-ΔtasA	3.6×10^{-5}	1.5×10^{-5}
B310mA	7.9×10^{-5}	3.6×10^{-5}
WT (pellicle)	4.2×10^{-5}	2.5×10^{-5}

E.

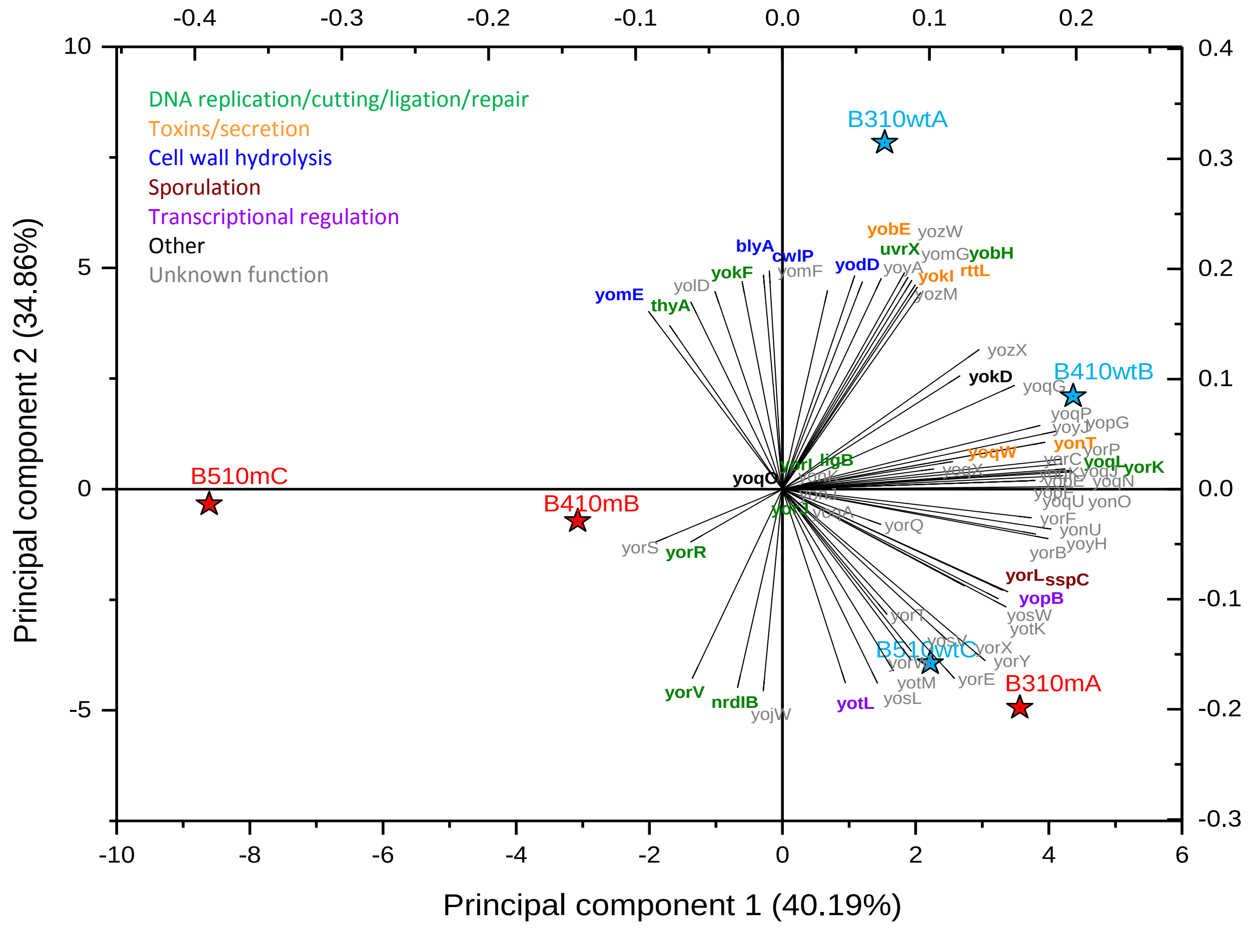

Supplementary Figure 5. Comparisons of SNPs patterns and mutation rates in the eMP and eNMP strains. A. The SPβ prophage regions of 6 evolved populations (the above panel) and corresponding single isolates (the below panel) were aligned with the SPβ sequence of the ancestor strain using VISTA tools (http://genome.lbl.gov/vista/index.shtml, 54). Peaks represent non-conserved regions based on scores for each base pair in the genomic interval of 100-bp and conservation identity 70%. A region is considered conserved if the conservation over this region is greater/equal to 70% and has minimum length of 100-bp. **B.** Percent distribution of different SNPs types detected in 6 evolved single isolates. Non-synonymous similar substitutions had Blosum62 matrix score ≥1. Non-synonymous not similar substitutions had Blosum62 matrix score ≤ 0. **C.** Fluctuation assay (n=10) was done to determine the frequency of streptomycin-resistant mutants in Δ*eps*-Δ*tasA* ancestor, evolved mutant B410mB, evolved WT B310wtA and WT ancestor strains in liquid and in pellicle (P). Analysis was conducted using LB agar medium without and with 50µg/ml streptomycin. Results show overlapping frequency rate of evolved strains with the ancestor wild type. Mutation rate estimates was determined using bz-rates web based tool (http://www.lcqb.upmc.fr/bzrates). Data points represent the mean and bars span from lower to upper CI (95%). **D.** Linear plots showing the number of SNPs per each mutated gene (genes are sorted according to their functional group) for eMPs (shown in blue) and eNMPs (shown in red). **E.** PCA biplot containing the mutated genes of eMPs (B310wtA, B410wtB and B510wtC) and eNMPs (B310mA, B410mB and B510mC) strains in two

dimensions using their projections onto the first two principal components. All mutated genes are represented on the plot using their weights for the components. The genes are color-coded by functional category. The scales shown on x and y axis are for the strains; the scales shown above the plot and on the right are for genes.

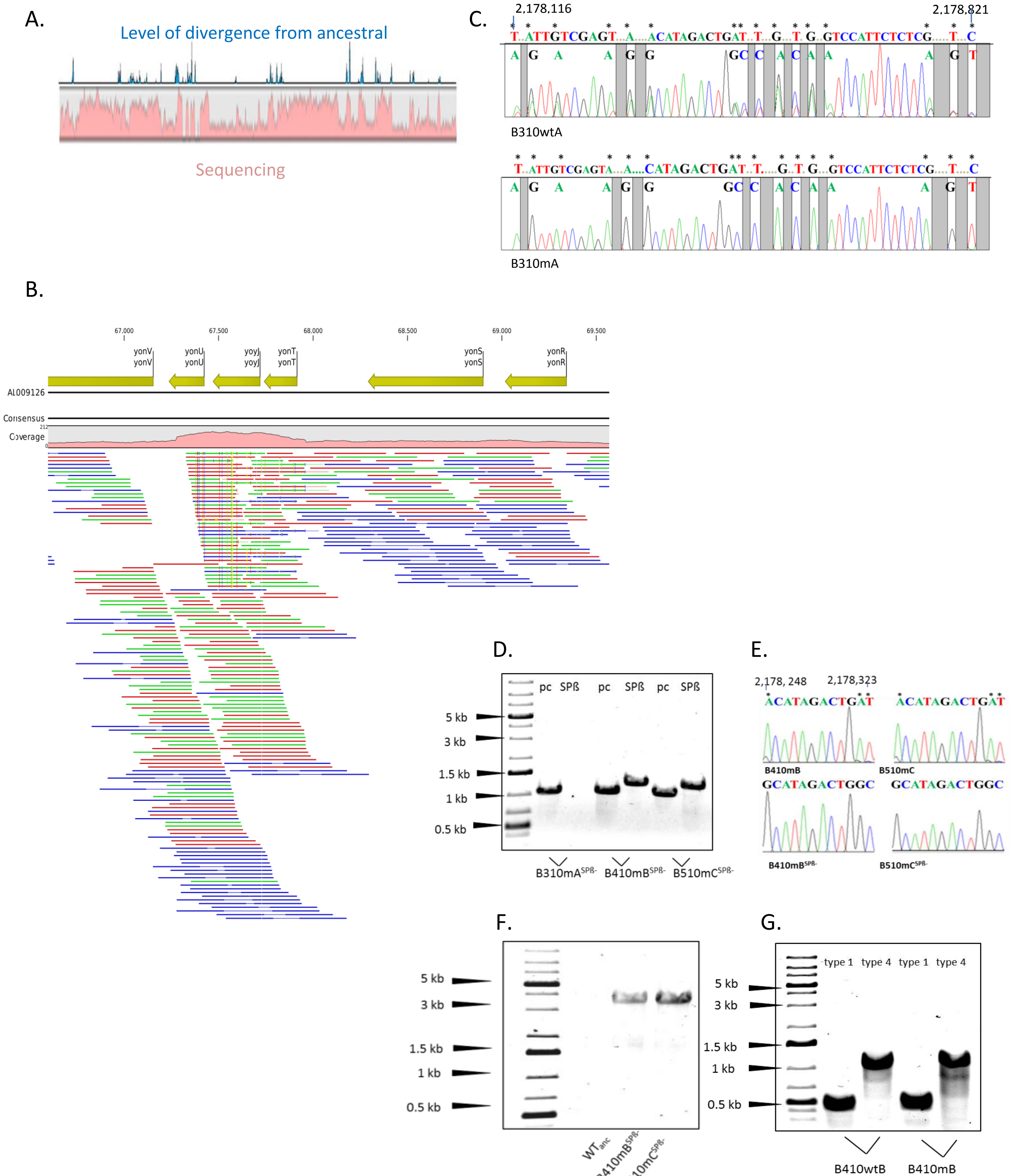

Supplementary Figure 6. Detailed molecular analysis confirm presence of genome rearrangements in the evolved strains. A. Alignment of Vista curve (54) showing regions of SNPs accumulation within the evolved SPβ sequence (see also S5A) (blue) and the sequencing coverage curve (pink) indicates duplications of mutated SPβ fragments. **B.** Sequence reads obtained for the evolved *B. subtilis* strains that are mapped to reference genome. Color coding: green-reads mapped based on the FWD strand; red-reads mapped based on the REV strand; blue–paired reads. Mismatch SNPs are marked as vertical stripes. Two types of reads were identified: reference-matching reads without SNPs and reads with SNPs. **C.** Selected SPβ fragment was amplified by PCR using B310wtA and B310mA gDNA and re-sequenced by Sanger method. Product obtained from B310wtA showed double peaks on the sequencing chromatogram at the positions of SNPs detected by high-throughput sequencing. The double peaks correlated with ~50% SNPs frequency in SPβ region, confirming presence of two types of SPβ fragments in the B310wtA, but not in the B310mA. **D.** Presence of a selected SPβ fragment was confirmed by PCR using primer pair oTB86F/oTB87R (for oligo sequences, see Supplementary Table 3) in the strains B410mB ΔSPβ and B510mC ΔSPβ but not in the B310mA ΔSPβ. Pc= positive control, amplified fragment outside SPβ. **E.** Comparison of Sanger sequencing chromatograms obtained for selected SPβ fragments before and after SPβ deletion in B410mB and B510mC strains. After deletion of SPβ PCR product could still be obtained (see Fig. S6D), but it produced clear sequencing result with single peaks at the SNPs positions containing solely the evolved sequence. **F.** Knockout of SPβ prophage region in strains B410mB ΔSPβ and B510mC ΔSPβ was confirmed by PCR using primers oAD1F/oAD2R. **G.** Presence of predicted genome rearrangements (see Fig. 5CD) of type 1 and type 4 was confirmed in the evolved strains and not in the ancestor strains using primer pairs AD4F/oAD5R and oAD6F/oAD7R, respectively.

A.

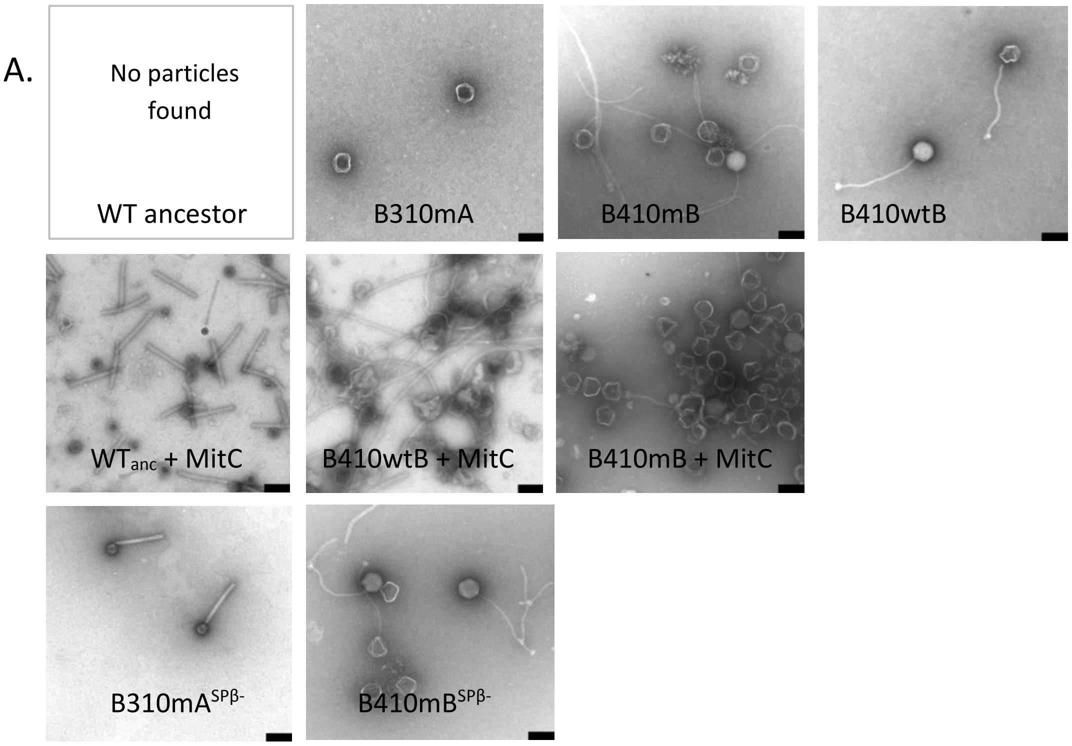

B.

Lawn ↓ / Supernatant donor →

	WT anc	Mut anc	B310wtA	B410wtB	B510wtC	B310mA	B410mB	B510mC	B310mA SPß-	B310mA SPß-	B310mA SPß-
WT anc			++	++		+++	++++	+++		++++	+++
Mut anc			++	+		++	+++	++		+++	+++
B310wtA											
B410wtB											
B510wtC			+	+		+	+	+		+	+
B310mA											
B410mB											
B510mC											
B310mA SPß-			+++++	++++	++	+++	++++	+++		+++	+++
B310mA SPß-			++				+				
B310mA SPß-											

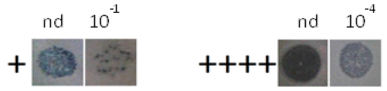

C.

Lawn ↓ / Supernatant donor →

	B110	B210	B310	B410	B510	A110	A210	A310	A410	A510
WT anc	++++	+	++++	++++	++++		++++			

Supplementary Figure 7. Evolved strains spontaneously release SPbeta-like phage that is lytic towards the ancestral strains. A. Electron micrographs of phage particles purified from *B. subtilis* supernatants. No phage particles were spontaneously released by the WT ancestor strain. All evolved strains tested spontaneously released SPβ-like phage particles and treatment with Mitomycin C dramatically increased the number of those particles. WT ancestor incubated with Mitomycin C and the evolved B310mA ΔSPβ produced solely PBSX-like phage particles. Scale bar equals 100 nm. **B.** Results of the plaque assays performed with the ancestor WT, ancestor Δ*eps*-Δ*tasA* and all the evolved strains, where each strain served both as a supernatant donor and as a potential host. To better access lytic activity of the strains, their supernatants were diluted using saline solution and scored as shown on the scale below. Blank cell in the table translates into lack of lytic activity towards given host even if non-diluted supernatant was applied on the loan. **C.** Lytic activity of all evolved populations (transfer method A and transfer method B) against the WT ancestor was tested. All populations that showed increase of Δ*eps*-Δ*tasA* ratio (see Fig. 2) thorough the evolutionary time showed lytic activity towards the WT ancestor.

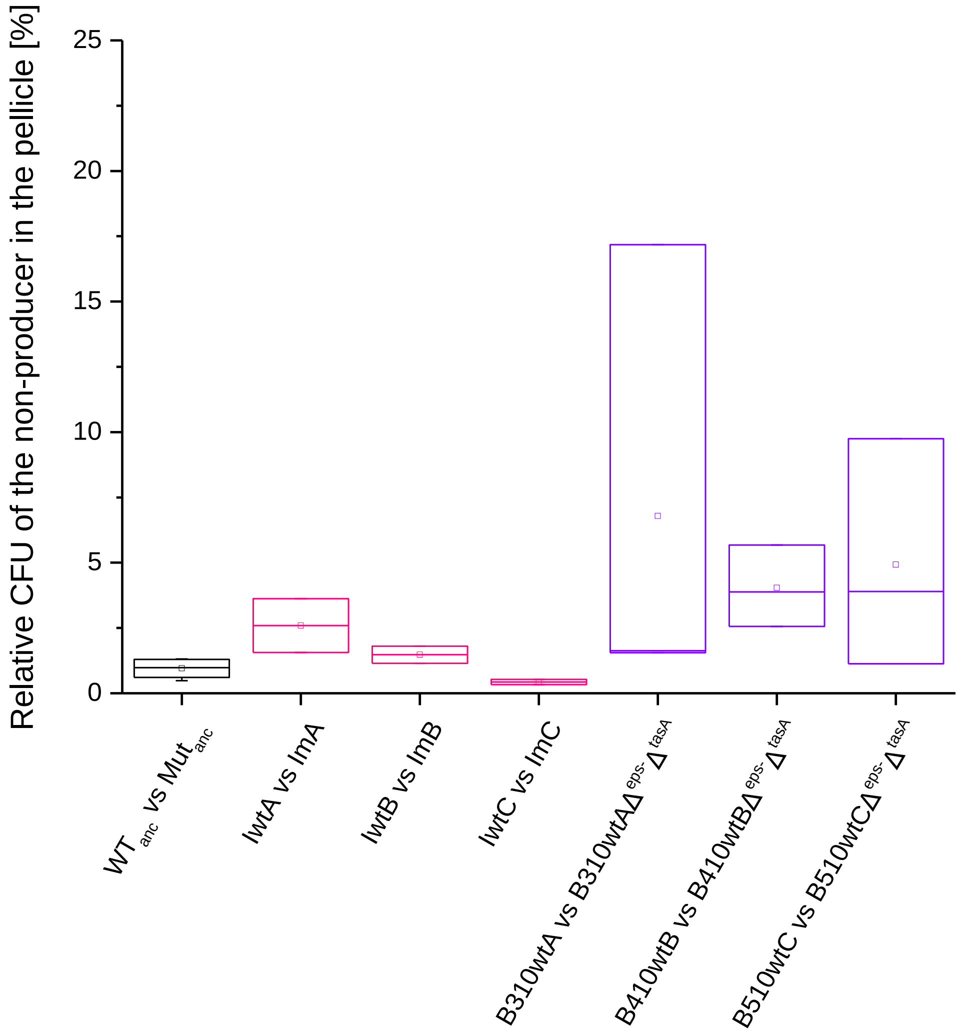

Supplementary Figure 8. When producers and non-producers carry the same evolved genetic background, improved incorporation of non-producers is not observed. Pellicle competition assay performed for 3 randomly selected WT and Δ*eps*-Δ*tasA* colonies after the infection assay (n=2) (columns 2-4). In addition, pellicle competition assays between WT evolved strains and their corresponding strains with Δ*eps*-Δ*tasA* markers (n=3) were performed (columns 5-7). Boxes represent Q1-Q3 and lines represent median values. Results show that the infected mutant strains cannot increase their performance when competed against infected WT strains. The same is true for the evolved WT strains with deleted *eps* and *tasA* that behave comparably as the ancestor Δ*eps*-Δ*tasA* mutant when competed with WT (first column). The experiments were performed twice.

Supplementary Table 1. Strains used in this study

Strain name	Genotype	Reference
168	Δ*trpC*	[1]
DL821	NCIB 3610 *lacA::*P_{tapA}*:yfp* (Mls^R)	[2]
DL1032	NCIB 3610 Δ*epsA-O*::Tet^R Δ*tasA*::Km^R *amyE::*P_{srfAA}*-lacZ*	[2]
168 hymKATE P_{tapA}-*yfp*	Δ*trpC amyE:* $P_{hyperspank}$-*mKATE* (Cm^R) *lacA::*P_{tapA}*:yfp* (Mls^R)	this work
Δ*eps*-Δ*tasA*	Δ*trpC* Δ*epsA-O*::Tet^R Δ*tasA*::Km^R	this work
Δ*eps*	Δ*trpC* Δ*epsA-O*::Tet^R	this work
Δ*tasA*	Δ*trpC* Δ*tasA*::Km^R	this work
168 hyGFP	Δ*trpC amyE*::$P_{hyperspank}$-GFP (Cm^R)	[3]
168 hymKate	Δ*trpC amyE*::$P_{hyperspank}$--mKATE2 (Cm^R)	[3]
Δ*eps*-Δ*tasA* hyGFP	Δ*trpC* Δ*epsA-O*::Tet^R Δ*tasA*::Km^R *amyE:*$P_{hyperspank}$-*GFP* (Cm^R)	this work
Δ*eps*-Δ*tasA* hymKate	Δ*trpC* Δ*epsA-O*::Tet^R Δ*tasA*::Km^R *amyE:*$P_{hyperspank}$-*mKATE* (Cm^R)	this work

Supplementary Table 2. Evolved populations and single isolates obtained in the study and used for further analyses.

Co-culture name	Population	Single isolate	Strains derived from single isolates
B310	B310wt	B310wtA	B310wtA GFP
			B310wtA mKate
			B310wtA Δ*eps*Δ*tasA*
		B310mB	
		B310mC	
	B310m	B310mA	B310mA GFP
			B310mA mKate
			B310mA ΔSPß
		B310mB	
		B310mC	
B410	B410wt	B410wtB	B410wtB GFP
			B410wtB mKate
			B410wtB Δ*eps*Δ*tasA*
		B410wtA	
		B410wtC	
	B410m	B410mB	B410mB GFP
			B410mB mKate
			B410mB ΔSPß
		B410mA	
		B410mC	
B510	B510wt	B510wtC	B510wtC GFP
			B510wtC mKate
			B510wtC Δ*eps*Δ*tasA*
		B510wtA	
		B510wtB	
	B510m	B510mC	B510mC GFP
			B510mC mKate
			B510mC ΔSPß
		B510mA	
		B510mB	

Supplementary Table 3. Primers used in this study

Primer	Experimental purpose	Sequence
oAD1F	confirm SPbeta deletion from the original position in the chromosome	ATCTGGACTGGCACCTTATGGATACC
oAD2R	confirm SPbeta deletion from the original positions in the chromosome	CTGCTCTGGAAAGGAAGGCAGAGTAA
oTB86F	check for additional copy of SPbeta, examine Sanger chromatogram	CACGCTTGTCTCCAAACC
oTB87R	check for additional copy of SPbeta, examine Sanger chromatogram	GATTGGCCATAACAGACC
oAD4F	confirm SPbeta rearrangement type 1	CTAAGGAGAGATAGGGCAT
oAD5R	confirm SPbeta rearrangement type 1	TGGTTTGAAGGCCATCACA
oAD6F	confirm SPbeta rearrangement type 4	CGATTTCAGCTGCCAAATCC
oAD7R	confirm SPbeta rearrangement type 4	CAGGAAAACTGGTCAGAAAC
oTB74	confirm *eps* deletion	GGGAAGTGCAGTAAATTAG
oTB75	confirm *eps* deletion	GAAACGGATTCAGCATTTAG
oTB73	confirm *tasA* deletion	GATCAGCAGCGCCATTAGAG
oTB72	confirm *tasA* deletion	CATGGCATGCGCCTGAGCAGAGGCACTAAC

Supplementary References:

1 Kovács, Á. T. & Kuipers, O. P. Rok regulates *yuaB* expression during architecturally complex colony development of *Bacillus subtilis* 168. *J Bacteriol* **193,** 998-1002 (2011).
2 López, D., Vlamakis, H., Losick, R. & Kolter, R. Paracrine signaling in a bacterium. *Genes Dev* **23,** 1631-1638 (2009).
3 van Gestel, J., Weissing, F. J., Kuipers, O. P. & Kovács, Á. T. Density of founder cells affects spatial pattern formation and cooperation in *Bacillus subtilis* biofilms. *ISME J* **8,** 2069-2079 (2014).
